# Genome Sequence of a New Carnation Small Viroid-Like RNA, CarSV-1

**DOI:** 10.1128/mra.01219-22

**Published:** 2023-02-22

**Authors:** Timo M. Breit, Wim C. de Leeuw, Marina van Olst, Wim A. Ensink, Selina van Leeuwen, Rob J. Dekker

**Affiliations:** a RNA Biology & Applied Bioinformatics Research Group, Swammerdam Institute for Life Sciences, Faculty of Science, University of Amsterdam, Amsterdam, The Netherlands; Katholieke Universiteit Leuven

## Abstract

Here, we report the genome sequence of a new circular viroid-like RNA (CarSV-1) derived from Dianthus caryophyllus (carnation) leaves. The CarSV-1 genome has notable sequence similarity (62%) to the well-studied CarSV viroid-like RNA and comprises the complete hammerhead consensus sequences involved in self-cleavage. CarSV-1 co-occurs with carnation viruses, such as CarMV.

## ANNOUNCEMENT

Viroids are a class of infectious plant pathogens consisting of a non-protein-coding small circular RNA genome (<400 nucleotides [nt]), without a protein coat (1). Small viroid-like RNAs are structurally similar to viroids and frequently function as plant virus satellites. For a genomics project undertaken with a Dutch plant-breeding company, we sequenced small RNAs from eight carnation plants (a commercial cultivar) collected from the company’s greenhouse facility. Six plants were positive by enzyme-linked immunosorbent assay (ELISA) for *Carnation mottle virus* (CarMV^+^), and two plants were uninfected (Table 1). The presence of (un)known viruses and viroids was investigated by means of the plant’s small interfering RNA (siRNA) response to viral and viroid infection (2). All investigated plants contained siRNAs from several known viruses (Table 1) and siRNAs related to an apparently new viroid-like RNA.

**TABLE 1:** Sequence read counts representing virus- and viroid-like RNA-related siRNAs

Type of RNA	Name^*b*^	GenBank accession no.	No. of reads in sample (CarMV ELISA result):^*a*^							
			E1-S01 (+)	E1-S02 (−)	E1-S03 (+)	E1-S04 (−)	E1-S05 (+)	E1-S06 (+)	E1-S07 (+)	E1-S08 (+)
Virus	CarMV	NC_001265.2	249,860	53	257,308	132	407,637	350,468	337,198	264,719
	AltMV	NC_007731.1	15,665	5	31,027	4	21,259	16,895	20,458	30,164
	CLV	MN450069.1	0	2	9,867	1	0	0	5,477	0
	CCV-3 (RNA1)	NC_034513.1	486	275	334	2,039	184	214	161	381
	CCV-3 (RNA2)	NC_034523.1								
	CERV	NC_003498.1	0	0	0	0	1	0	0	0
Viroid-like RNA	CarSV	X68034.1	107,153	22	109,447	37	74,024	62,442	84,008	104,935
	CarSV-1	OP506135.1	64,706	7	60,517	21	36,885	41,622	45,282	64,703
Total no. of reads (millions)			5.3	3.3	4.0	3.8	4.0	3.9	3.9	3.5

a No. of reads per million total reads.

b AltMV, *Alternanthera mosaic virus*; CLV, *Carnation latent virus* (or *Carlavirus*); CCV-3, *Carnation cryptic virus 3*; CERV, *Carnation etched ring virus*; CarSV, carnation small viroid-like RNA.

Small RNA was obtained by grinding flash-frozen ~1-cm^2^ leaf fragments into powder using a mortar and pestle and dissolving the powder in QIAzol lysis reagent (Qiagen) for RNA isolation and small RNA enrichment using the miRNeasy minikit (Qiagen). Barcoded small RNA transcriptome sequencing (RNA-seq) libraries were generated using the Ion total RNA-seq kit v2 and Ion Xpress RNA-seq barcoding kit (Thermo Fisher Scientific) and sequenced (150 bases; Ion Proton system, Ion PI Hi-Q Chef kit, Ion PI v3 chips; Thermo Fisher Scientific). Adapter and quality trimming were performed using Torrent Suite v5.8.0 software (default parameters), resulting in 3.3 to 5.3 million reads per sample (Table 1). All reads over 18 nt that passed the default Ion Proton quality standards were used.

To detect known viruses or viroids, the sequencing reads were mapped using the standard BLAST algorithm to the complete NCBI virus/viroid database. siRNAs related to three known viruses were found in all CarMV^+^ plants, whereas the two CarMV**^−^** plants only contained cryptic virus-related siRNAs (Table 1).

**FIG 1:**
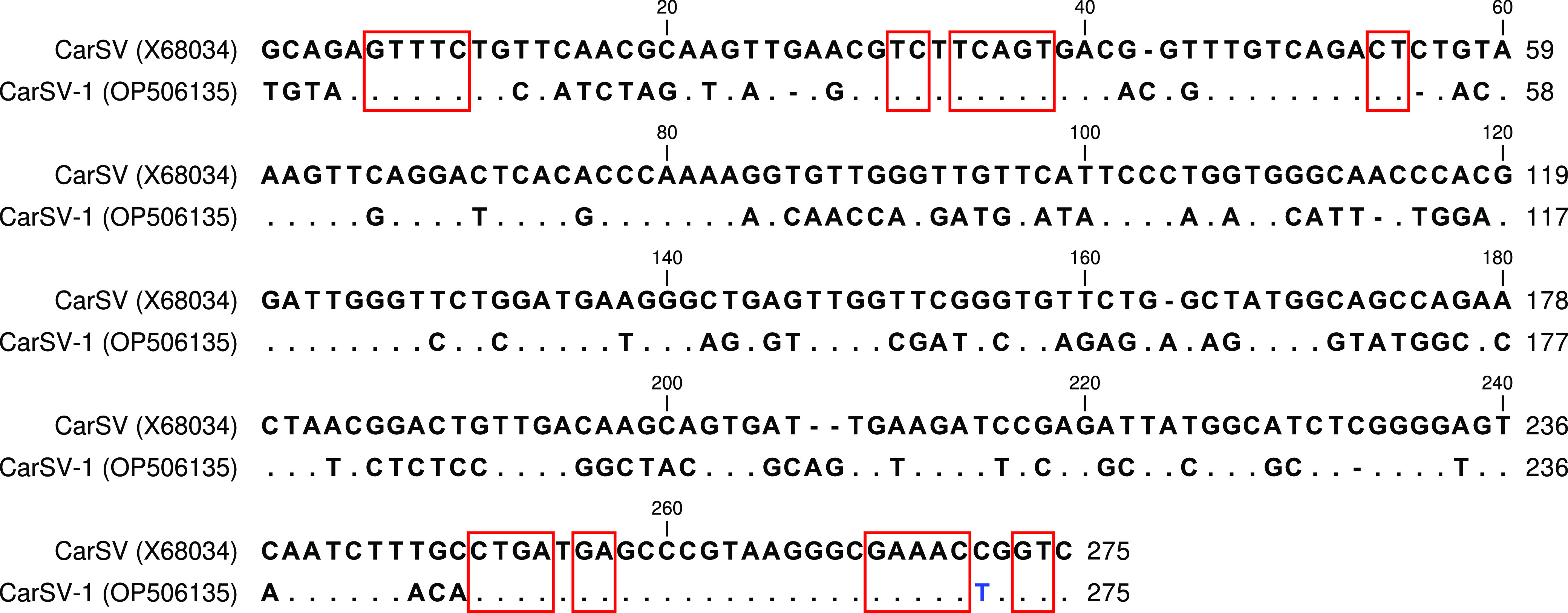
Sequence alignment of the viroid-like RNA genomes of CarSV and CarSV-1. The conserved nucleotides for the hammerhead structures of the plus and minus strand viroid-like RNAs are indicated with red boxes. In two out of the six samples, the T (blue) at position 271 was a C.

To identify potential new viruses and viroids, small RNA reads that did not map to known virus or viroid sequences were assembled per sample using the SPAdes v3.14.1 genome assembler (3) with default parameters. Most of the resulting contigs were related to carnation genome sequences, but in the CarMV^+^ samples, there was one contig that showed sequence similarity to carnation small viroid-like RNA (CarSV; GenBank accession number X68034) (4–7). This siRNA-derived contig sequence appears to refer to a circular RNA, as its ends were identical (data not shown). Removal of the duplicate sequences resulted in a 275-nucleotide sequence with an average coverage depth of 6,674× per CarMV^+^ sample. This sequence showed substantial sequence similarity (62%) to the CarSV sequence and had an identical GC content (51%). Furthermore, it contained the complete hammerhead consensus sequences for both the plus and minus RNA strand forms (Fig. 1) (4, 6); hence, we named it CarSV-1. Plants infected with CarSV can either be symptomless or be affected by a growth abnormality consisting of extensive shoot proliferation (8).

Reverse transcriptase quantitative PCR (RT-qPCR) analysis using a CarSV-1-specific primer/probe combination (forward primer, GTGTAGATTCCAGGCTACGGC; reverse primer, TGCCACGTAGCCTCAAGG; probe, TTGGGTCCTCGATGATGGGA) confirmed the presence of CarSV-1 RNA in the CarMV^+^ samples, with threshold cycle (*C_T_*) values of 16 to 18 cycles, as has been observed for CarSV (8). In the control samples S02 and S04, CarSV-1 was not detected by RT-qPCR. Whereas CarSV has been affiliated with *Carnation etched ring virus* (CERV) (7, 9), CarSV-1 might be associated with CarMV or *Alternanthera mosaic virus* (AltMV), based on the observed co-occurrence in this study.

### Data availability.

The raw sequence reads have been deposited in the NCBI Sequence Read Archive under BioProject accession number PRJNA885393. The genome nucleotide sequence of CarSV-1 has been deposited under GenBank accession number OP506135.
